# Perspectives from the Global South inform and affirm the contemporary river conservation paradigm: A commentary on Feio et al.

**DOI:** 10.1111/gcb.16474

**Published:** 2022-10-19

**Authors:** Charles B. van Rees

**Affiliations:** ^1^ Odum School of Ecology & River Basin Center University of Georgia Athens Georgia USA

The equitable and sustainable management of rivers is among the biggest environmental challenges of the Anthropocene. As sources of fresh water (itself essential for nearly all human enterprise), energy production, wastewater conveyance, and diverse cultural uses and values, rivers are vitally important ecosystems (Brauman et al., 2014; Yeakley et al., 2016). However, the intense exploitation of these services, combined with hydrological shifts from a changing climate and extensive management to reduce flood risk, has led to widespread declines in their ecosystem condition and in riverine biodiversity (Albert et al., 2021; Vörösmarty et al., 2010). With multiple additional stressors on rivers like invasive alien species and water quality impacts from upland land use change, the scientific community has focused on biotic indices for monitoring riverine health. The use of such indices to describe the ecological condition of a river system, termed *bioassessment* or *biomonitoring*, is intended to provide an integrated perspective of the multiple potential stressors acting simultaneously on the whole ecosystem (Feio et al., 2021). By directly assessing the diversity and abundance of focal taxonomic groups, bioassessment programs provide important perspective into the quality and potential service delivery of a river system, but also a proximate measure of the local state of biodiversity. Given trends over time, such data can answer important questions about the efficacy of conservation policy, ecological restoration, and the management of biodiversity, supporting conservation evidence approaches.

Originating in the mid‐to‐late 20th century, the assessment of rivers via biological indices became codified into environmental laws and policies in countries like the United States, Canada, New Zealand, and the governing body of the European Union. This created the opportunity for regional and global assessments of river ecosystem health. While the operational bioassessment programs in many wealthier, industrialized countries have supported important research for decades (e.g., Miltner & Rankin, 1998), industrializing, historically marginalized nations (also known as the Global South) are important “blind spots” for global freshwater conservation, with fewer available data (Alahuhta et al., 2019). This is despite the fact that many of these nations contain biodiversity hotspots, and will see growing ecological impacts from sweeping infrastructure development plans and growing societal demands for resources in coming decades (e.g., Ng et al., 2020). Furthermore, the general scientific community may have limited access to available biomonitoring data from Global South countries due to its marked reliance on the English language, a factor that greatly limits global conservation applications (Amano et al., 2021).

The research paper by Feio et al. (2022) in this issue describes an exciting and important effort to collate river bioassessment data worldwide, with special attention to available datasets in the Global South. Importantly, this undertaking involved co‐authors from nations like China, Nepal, Brazil, Bolivia, Nigeria, and South Africa. By gathering available bioassessment data on macroinvertebrates and fishes from 45 countries on six continents, they built a unique database with coverage from regions that are under‐represented in the scientific literature. Feio and colleagues reclassified indicator metrics from these diverse bioassessment protocols into three broad categories: Good, Impaired, and Severely Impaired. Importantly, Feio and colleagues also assessed the degree of influence of social and ecological variables on the proportion of survey sites with different condition rankings for each region.

Among a multitude of thought‐provoking findings, several are worthy of emphasis here, and call for further inquiry by the larger freshwater conservation community. First, Feio and colleagues' work corroborates the contemporary understanding that freshwater ecosystems worldwide are intensely threatened with the sobering finding that at most half of river systems are currently in good ecological condition according to macroinvertebrate indices, and even less than that when considering fishes. Furthermore, nearly one in three river systems were found to be severely impaired. Lower quality ratings among fish‐derived indices were more frequent in heavily dammed rivers, even when invertebrate‐derived indices indicated higher quality, highlighting the importance of connectivity for declining fish species at the global scale.

Notably, biological condition among both sets of metrics was more closely tied to variables pertaining to environmental legislation than to the duration of industrialization in a region. This lends credence to the efficacy of environmental legislation and may suggest that large‐scale restoration projects in damaged riversheds may yield tangible ecological benefits. Land management variables like watershed forest cover and freshwater protected areas also correlated with higher quality. This, in turn, speaks to the effecticacy of forest conservation, upland management, and strategic habitat protetion in freshwater biodiversity conservation.

Feio and colleagues also found especially low biological quality in arid and semi‐arid regions, which speaks both to the hydrological sensitivity of freshwater ecosystems in dry landscapes, but also to the potential impact of anthropogenic water demand on river systems. The increasing severity of droughts and rising water demand in many drier regions puts additional pressure on extent riverine ecosystems. The role of such competing demands for water in biodiversity declines is well studied for basins in wealthier countries like Australia, but may have different dynamics in understudied regions in the Global South, whose economic, cultural, and ecological contexts could be vastly different. Increased attention to the interactions of biodiversity conservation and national water security will be important in confronting these challenging socio‐ecological circumstances (van Rees et al., 2019).

This work provides much‐needed support for the efficacy of environmental legislation in protecting riverine ecosystems, which we may hope will dispel doubts about its utility in sustainable water governance. Furthermore, Feio et al. (2022)’s great effort toward gaining ecological insights from the Global South should serve as an example to future studies seeking a global perspective in biodiversity conservation. An overdependence and overemphasis on a few wealthy countries leads to a highly distorted vision of the world's ecosystems and sustainability challenges. Further efforts to counteract this bias are commendable and necessary for equitable and effective conservation science.

## Data Availability

Data sharing is not applicable to this article as no new data were created or analyzed in this study.
